# Assessment of Premutation in Myotonic Dystrophy Type 1 Affected Family Members by TP-PCR and Genetic Counseling

**DOI:** 10.1155/2014/289643

**Published:** 2014-02-23

**Authors:** Ashok Kumar, Sarita Agarwal, Sunil Pradhan

**Affiliations:** ^1^Department of Genetics, Sanjay Gandhi Post Graduate Institute of Medical Sciences, Lucknow 226014, India; ^2^Department of Neurology, Sanjay Gandhi Post Graduate Institute of Medical Sciences, Lucknow 226014, India

## Abstract

Myotonic dystrophy type 1 (DM1) is caused by the expansion of an unstable CTG repeat located in the 3′-UTR of (*DMPK*) the *DM protein kinase* gene. Patients with DM1 have expansions of greater than 50 repeats and up to many thousands. In the present study we aimed to evaluate the utility of TP-PCR in diagnostics as well as the assessment of premutation carriers in proband families. Twenty-seven DM1 cases were enrolled (from twenty-six families) and the 13 families of these cases came forward for family screening. The patient group constitute 22 males and 5 females and the average age of onset was 32.8 years (range 17 to 52). All clinically diagnosed DM1 cases and their family members DNA samples were analyzed by TP-PCR. All the cases were found to be positive for the CTG repeat expansion. Among those five families, four had at least an asymptomatic carrier. In the remaining one family other than the proband none was found to be neither affected nor asymptomatic. We reconfirmed the utility of PCR based screening for DM1 as being reliable and rapid molecular test and it should be used as an initial screening test for all patients with DM and their family members for initial screening purpose.

## 1. Introduction

Myotonic dystrophy type 1 (DM1) is the most common form of adult muscular dystrophy, with an incidence of approximately 1 in 8,000. It is an autosomal dominant disorder characterized by myotonia, progressive muscle weakness and wasting, cataracts, hypogonadism, frontal balding, cardiac conduction defects, and cerebral involvement [1, 2]. Out of four types of myotonic dystrophy, myotonic dystrophy types 1 and 2 (DM1 and DM2) are most common and caused by mutation in two different genes *DMPK *(*Dystrophia myotonica protein kinase*) and cellular nucleic acid-binding protein, that is, *CNBP* (previously known as *ZNF9,* that is, *Zinc finger protein* [3, 4]). The severity of the disease is highly variable, with the phenotypic spectrum ranging from asymptomatic or very mildly affected adults to severely affected neonates with the congenital form of the disease. Congenital DM1 is characterized by severe hypotonia and respiratory distress and often results in early death [5]. Polyhydramnios and poor fetal movements precede the birth of an infant with congenital DM1 [6]. The affected parent is nearly always the mother and congenital DM1 occurs in a quarter of offspring of affected DM1 mothers [7].

DM1 is caused by the expansion of an unstable CTG repeat located in the 3′-untranslated region of the *DM protein kinase *(*DMPK*) gene [8–13]. The number of CTG repeats is polymorphic in the general population, with a range of 4 to approximately 37 repeats (Table 1; see [9, 12–16]). Patients with DM1 have expansions of greater than 50 repeats and up to many thousands. Size of the repeat is positively correlated with severity of the disease and inversely correlated with age of onset of symptoms [11, 14, 17, 18]. The frequent increase in length of the expanded alleles upon transmission through the germline underlies the anticipation seen in families with DM1 [19].

DM1 mutation can be carried by asymptomatic or minimally affected individuals who have relatively small expansions, termed premutations, in the range of approximately 50 to 80 copies [20]. Although premutations can be inherited relatively stably for several generations if transmitted by women [20, 21], passage through the male germline almost invariably results in a large increase into the full disease range [20]; [22]. Therefore, it seems unlikely that the DM1 mutation could be stably maintained in the population for any significant length of time in this state. The boundary between normal, stable alleles and disease-associated, unstable alleles has not been precisely defined because of the paucity of alleles in the 38 to 50 repeat ranges. This class of alleles, called premutation [23], forms the pool from which novel premutations and DM1 full mutations most likely arise, for which predictive testing can be offered to a large number of at-risk people. Guidelines for direct predictive genetic testing recommend the application of DNA analysis to those diseases where the risk prediction may be followed by treatment. Although till date no effective treatment is available for DM, both medical interventions and preventive measures can improve the patient's condition [24]. In the present study we aimed to evaluate the utility of triple primed polymerase reaction (TP-PCR) in diagnostics as well as for assessment of premutation carriers in proband families keeping in view of both early medical interventions and preventive measures.

## 2. Materials and Methods

Blood samples were obtained from Indian patients (clinically diagnosed as DM1) and their family members from Department of Neurology, SGPGIMS, Lucknow. Informed consent was obtained from all family members after the nature and possible consequences of the study were explained. Genomic DNA was isolated from standard phenol chloroform method.

TP-PCR (triplet primed-polymerase chain reaction)

Screening for CTG repeat expansion was performed using PCR primers spanning the CTG repeat in 3′ UTR region of the *DMPK* gene [25]. TP-PCR assay was carried out in a reaction volume of 25 *μ* litre with P1, P2, P3, and P4 (P1, P2, and P3 are flanking primers and P4 is internal primer) primers in the concentration of 10 pmol each along with 50 ng of genomic DNA, 1 U of Taq polymerase, and 200 *μ*M of dNTPs. The temperature profile adopted in TP-PCR cycling was 4 min at 94°C followed by 30 cycles of 94°C for 1 min, 60°C for 1 min, and 72°C for 2 min and one cycle of 72°C for 10 minutes. The final products were analyzed on 2% agarose gel and segment analysis was done by ABI-310 Genetic Analyzer by the use of polymer (POP-4), Liz-500, and Hi-di formamide.

## 3. Results

**Figure 1:**
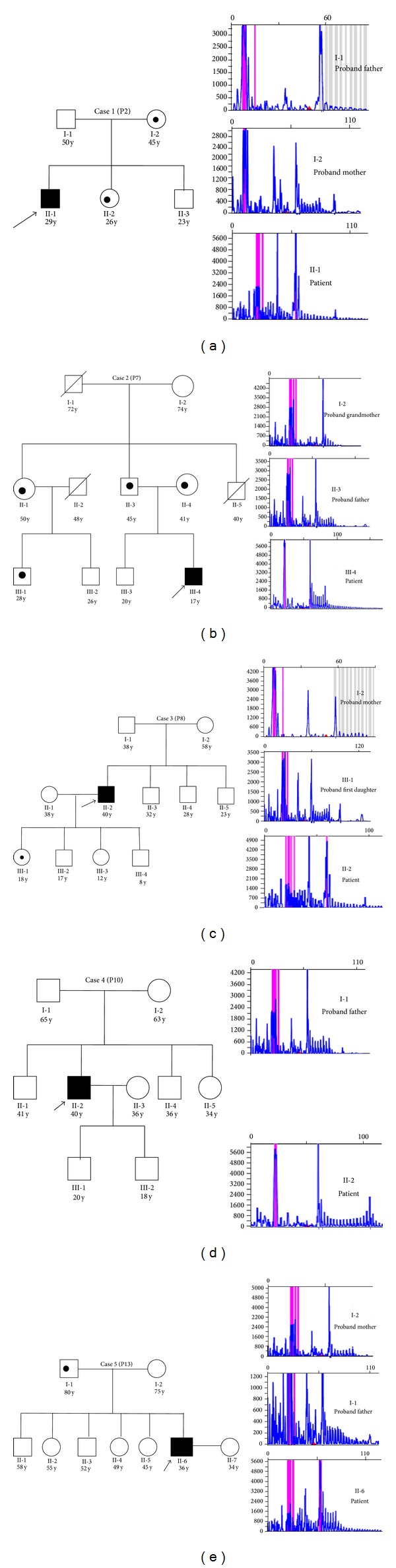
(a) Case 1 (P2): I-1, I-2, II-2, and II-3 were proband's father, mother, younger sister, and younger brother, and II-1 was DM1 patient. Square = male; circle = female; black symbol = patient (II-1); and black dots in symbols represent asymptomatic carriers (proband's mother, I-2, and younger sister, II-2). (b) Case 2 (P7): I-2, III-2, and III-3 all were normal and II-1, II-3, II-4, and III-1 were asymptomatic carriers. (c) Case 3 (P8): II-2 was DM1 patient and III-1 (first daughter) was asymptomatic carrier for DM1. (d) Case 4 (P10) screening: all relatives of the proband were normal for disease. (e) Case 5 (P13): proband father (I-1) was asymptomatic while all were normal for disease.

From 2011 to 2013, we carried out genetic screening in 27 cases of DM1 and their seventy-five family members (from twenty-six families) referred by Department of Neurology. There were 22 males and 5 females and the average age of onset was 32.8 years (range 17 to 52 years). After genetically confirming the DM1 in probands, we communicated the family members under extended genetic screening service. The complete family screening was done in thirteen families and in the rest of cases we had only proband and an escort. Here we are providing the screening details of only five families. The clinical manifestations and the repeat size of the proband (patient) are provided in Table 2. Figures 1(a)–1(e) and Table 3 represent five pedigrees where full molecular testing along with their family members was performed.

*Case  1* (P2). A Brahmin male patient with the age of onset of 29 years presented to OPD with a complaint of muscle wasting in the hands, profound facial weakness, and mild mental retardation. Molecular testing of patient confirmed CTG repeat expansion. Further family screening established the fact that his father was absolutely normal; both mother and sister were asymptomatic. The family members have not given the consent for analysis of another 23-year-old male child (Table 3, Figure 1(a)) in spite of providing genetic counseling.

*Case  2* (P7). A 17-year-old male child from Vaishya family was referred to our department for molecular testing with a history of grip loss, tongue slippery, and high salivation during sleep for the previous 4 years. Clinical examination confirmed muscle wasting in the upper arm and palm, jaw and temporal wasting, facial weakness, and hypersomnia. Screening of his family members like proband's father, mother, aunt (II-1), and his first cousin (III-1) showed that they were asymptomatic carriers, while proband second cousin (III-2), elder brother (III-3), and grandmother (I-2) were normal for CTG repeats, and grandfather (I-1) and uncles (II-2, II-4) died much earlier before the molecular testing of the disease (Table 3, Figure 1(b)) though clinically diagnosed as DM1.

*Case  3* (P8). A 40-year-old male Brahmin adult presented to Neurology OPD with severe form of DM with the complaints of muscle wasting in the legs and hands, jaw and temporal wasting, mental retardation, and diabetes. He was experiencing dyspepsia, dysphagia, and activity of daily living (ADL). On providing genetic counseling, the family members opted for molecular screening. Among the family members none of them were carriers except his first daughter (III-1; Table 3; Figure 1(c)). The family members had not given the consent for analysis of probandof another 8-year-old male child who was presently asymptomatic.

*Case  4* (P10). Case number 4 with DM at the age of onset of 40 years from Thakur community with complaint of muscle wasting, jaw and temporal wasting, and facial weakness was referred to the Department of Genetics for molecular evaluation. No clinical history of DM1 was recorded in family. All family members of the proband were found normal (Table 3) while the parents of the said family refused giving blood sample of another 34-year-old unmarried daughter (Figure 1(d)) for analysis. However, the genetic counseling was offered to them.

*Case  5* (P13). A Brahmin male proband of 36 years with difficulty in learning problem, speed, language problem, muscle wasting, jaw and temporal wasting, facial weakness, and mental retardation was reported in OPD of Department of Neurology. Once again no family history of DM was found, but the molecular screening of family members clearly denoted that his father was an asymptomatic carrier (Table 3; Figure 1(e)).

## 4. Discussion

Myotonic dystrophy (DM) is a common neuromuscular disorder comprising at least two genetically different forms. DM1 is caused by expansion of a (CTG)(*n*) repeat in the *DMPK* gene, while DM2 is caused by expansion of a (CCTG)(*n*) part of a complex repetitive motif (TG)(*n*)(TCTG)(*n*)(CCTG)(*n*) in the *ZNF9* gene [26]. DM1 is also a kind of progressive multisystemic autosomal dominant disorder with phenomena of anticipation; it denotes progressively earlier onset of the disease within successive generations. The disabilities are substantial and therefore early detection is mandatory for reproductive counseling of families in which the DM1 has been observed as they have 50% chances of each pregnancy to have an affected child.

Detection of the responsible expansions is complicated in both DM1 and DM2 because of the extremely variable length of the expanded alleles, which can contain even several thousands of repeats in both disorders [26]. In molecular methods, southern blotting, although used widely has a long laboratory turnaround time, is relatively expensive and has got hazards associated with the use of radioisotopes [27]. With respect to the genetic screening as well as diagnostics application, one of the commonly used detection approaches utilizes the combination of conventional PCR and triplet repeat-primed PCR [15, 25, 26, 28–31]. The TP-PCR can detect the presence of long allele size without determining the total size of the expansion or the exact number of expansions CTG. TP-PCR method is routinely used by western countries for the diagnosis of DM1 while in India DM1 is clinically diagnosed only.

The present study in five families screened by TP-PCR clarified that all the expanded alleles descend from the premutated alleles from respective parents and were subject of genetic counseling. However, in Case 4, though the expansion was of a sporadic event and the parents were not premutation carriers, the proband is affected and a source of transmitting the affected gene to his offspring. Such sporadic cases need to be counseled properly. Possibly, this may be due to the presence of noninterrupted repeat that increases repeat instability.

Therefore, DM1 is a disorder for which predictive testing can be offered to a large number of at-risk people. Guidelines for direct predictive genetic testing recommend the application of DNA analysis to those diseases where the risk prediction may be followed by treatment. Presently no effective treatment is available for DM; only medical interventions and preventive measures can improve the patient's condition [26]. We conclude that PCR based screening for DM1 is reliable and it should be routinely recommended as an initial screening program for all DM patients and their family members for counseling purpose and antenatal diagnostic purposes. If required, the method of southern blotting could be used for accurate sizing.

## Figures and Tables

**Table 1:** Range of CTG repeats in myotonic dystrophy type 1 (DM1).

Status	CTG repeat number
Normal	3–37
Carrier/premutation	38–50
Patient	>50

**Table 2:** Clinical and molecular profiles of probands.

Proband	Sex	Age of onset	Clinical feature	CTG repeat expansion (P: present, A: absent)
P1	Male	30	MW, JTW, FW, hypersomnia	P
P2	Male	29	MW, FW	P
P3	Female	45	MW, JTW, FW, hypersomnia	P
P4	Male	31	MW, JTW, FW, hypersomnia	P
P5	Male	30	MW, JTW, FW	P
P6	Male	23	MW, JTW, FW, hypersomnia	P
P7	Male	17	MW, JTW, FW, hypersomnia	P
P8	Male	40	MW, JTW, hypersomnia	P
P9	Female	40	MW, JTW, FW, hypersomnia	P
P10	Male	40	MW, JTW, FW	P
P11	Male	25	MW, JTW, FW	P
P12	Male	34	MW, JTW, FW, hypersomnia	P
P13	Male	36	MW, JTW, FW, hypersomnia	P

MW: muscle wasting; JTW: jaw and temporal wasting; and FW: facial weakness.

**Table 3:** Molecular profile of the proband family members.

Proband	Relation with proband	Repeat size	Status	Family history
Case 1 (P2)	Sister (26 y) Father (50 y) Mother (45 y)	40 15 35	Asymptomatic Normal Asymptomatic	No

Case 2 (P7)	Father (45 y) Mother (41 y) Aunt (50 y) First cousin (28 y) Second cousin (26 y) Elder brother (20 y) Grandmother (74 y)	35 40 50 50 10 20 10	Asymptomatic Asymptomatic Asymptomatic Asymptomatic Normal Normal Normal	No

Case 3 (P8)	Father (60 y) Mother (58 y) Wife (38 y) Brother 1 (32 y) Brother 2 (28 y) Brother 3 (23 y) Daughter 1 (18 y) Daughter 2 (12 y) Son 1 (17 y)	20 10 10 10 15 15 40 20 15	Normal Normal Normal Normal Normal Normal Asymptomatic Normal Normal	No

Case 4 (P10)	Father (65 y) Mother (63 y) Wife (36 y) Brother 1 (41 y) Brother 2 (36 y) Son 1 (20 y) Son 2 (18 y)	20 10 20 15 10 15 25	Normal Normal Normal Normal Normal Normal Normal	No

Case 5 (P13)	Father (80 y) Wife (20 y)	40 20	Asymptomatic Normal	No
